# Investigation of Sensor Component Properties for Application in DC Current Transformer

**DOI:** 10.3390/s26216813

**Published:** 2026-10-28

**Authors:** Jure Konjevod, Igor Štambuk, Marko Jurčević, Roman Malarić

**Affiliations:** 1 Department of Polytechnics, Dr. Franjo Tuđman Defense and Security University, Ilica 256b, 10000 Zagreb, Croatia; igor.stambuk@sois-ft.hr; 2 University of Zagreb Faculty of Electrical Engineering and Computing, Unska 3, 10000 Zagreb, Croatia; marko.jurcevic@fer.unizg.hr (M.J.); roman.malaric@fer.hr (R.M.)

**Keywords:** toroidal core, modular PXI instrumentation, current sensor, B-H curve, cage current shunt

## Abstract

Direct-current transformers (DCCTs) rely on small toroidal cores made of soft magnetic materials with high permeability, whose magnetic properties affect the sensitivity and linearity of the sensor. Reliable characterization of such small cores and of the resulting sensor component requires measurements with stated uncertainties, which are rarely reported together. This paper applies and integrates established measurement methods, namely, dynamic hysteresis loop measurements on ring specimens and dc current detection from the asymmetry of the saturation current peaks, into a laboratory test setup for the characterization of DCCT sensor components. Permalloy and nanocrystalline toroidal cores are characterized at 50 Hz. The nanocrystalline cores exhibit a maximum relative permeability of approximately 300 × 10^3^ and a coercivity of approximately 1.1 A/m, compared with approximately 100 × 10^3^ and 4.2–4.9 A/m for the permalloy cores. The sensor component with the tested core is calibrated against a reference current shunt for both current polarities, showing a linearity error of 0.59% FS in the range from −4 A to +4 A. The expanded uncertainty of the reference current measurement is ± 926 µA/A according to the GUM and ±906 µA/A using the Monte Carlo method (k = 2). The results show that the tested nanocrystalline cores are better suited for sensitive dc current detection and provide a basis for core selection and DCCT design.

## 1. Introduction

Magnetic properties characterization of specific materials is required to be known for specific sensor applications. The magnetic properties of nanocrystalline and permalloy toroidal cores as well as measurement test results for current sensors based on such toroidal cores are analyzed in this paper. Current sensors’ principles are usually divided in contact and contactless sensor principles [1]. Contact sensor principles most commonly imply using a shunt resistor while contactless current sensors rely on field effects of the measured current. Additionally, a more specific classification of current sensors usually consists of the following categories: current sensors based upon Ohm’s law of resistance, sensors that exploit Faraday’s law of induction, magnetic field sensors and so-called Faraday-effect-based current sensors, which exploit optical physics’ phenomena [2]. The focus of this paper is on the contactless sensor principle, i.e., on magnetic-field sensor type or more specifically, on the fluxgate sensor which exploits the nonlinear relation between the magnetic field, H, and magnetic flux density, B, within a magnetic material. The most common sensor based on the mentioned principle is a dc current transformer (DCCT) [3,4,5,6]. Regarding such sensors, the nonlinear magnetization of ferromagnetic materials is used for dc current determination. Thus, the substantial part of the sensor is its magnetic core. There are configurations with only one core, as in [7], but also with two, three or even four cores, as in [8]. However, the main working principle relies mostly on a pair of cores for dc component detection, which are driven with a modulator circuit in opposite phase, i.e., with a 180° phase shift [2]. Thus, the pair of cores ideally have equal characteristics in order to minimize the induced signal after subtraction, as proposed in [9]. Generally, soft magnetic materials which satisfy criteria for the sensor part in dc current transformers are crystalline, NiFe (Mo) alloy (permalloy), then amorphous, nanocrystalline, FeSiB alloy [9]. In this paper, two mentioned types of soft magnetic material cores, i.e., permalloy and nanocrystalline cores, are characterized with metrology-grade equipment. Nanocrystalline materials are more suitable for transformer structures in the higher-frequency area and almost form a rectangular hysteresis loop [10]. Such a nanocrystalline core was used as a sensor component in [11] as well.

In total, four cores are characterized and presented in this paper, i.e., two permalloy and two nanocrystalline cores, and all of them have the same geometrical properties, i.e., the same dimensions. Characterization of such magnetic cores usually implies a B-H curve, i.e., a hysteresis loop determination and first magnetization curve determination which is done in this paper as well. The measurement method to characterize toroidal cores is standardized in IEC 60404-6:2018 [12]. While IEC 60404-6 specifies the measurement method for ring specimens, it does not prescribe the instrumentation required when the specimen dimensions and windings are predefined and the resulting signals are in the millivolt range. Moreover, published studies of dc current sensors usually address the performance of the sensor, whereas the magnetic properties of the specific cores used and the performance of the sensor component built with them are rarely characterized by a complete evaluation of the referent setup and its measurement uncertainty [11,13]. Accordingly, the motivation for the development of a special setup to realize the measurement method from [12] was industry requirements, which often consist in conducting a number of control measurements to check the magnetic properties of the ordered sensor material with already predefined dimensions, number of windings, etc. In this paper, a ring core specimen’s dimensions are predefined as very small (Section 3) to be able to use medium-performance measurement equipment. Accordingly, it requires special metrology-grade equipment to precisely generate excitation signals and to acquire relevant output signals. In industrial practice, however, the magnetic properties of the ordered sensor material often have to be verified by a number of control measurements on cores with predefined dimensions and windings, which cannot be adapted to the measurement equipment. The cores used in DCCT sensors are a typical example: the ring specimens investigated in this paper have an outer diameter of 14.2 mm, an inner diameter of 12.1 mm and a height of 2 mm (Section 2), which gives a cross-sectional area of only 2.1 mm^2^. At the lowest magnetic field strengths of interest (about 0.1 A/m), the magnetizing current is only a few tens of microamperes, which corresponds to a voltage of only a few millivolts across the current-sensing resistor, while the induced secondary voltage is of the same order. Such signals cannot be measured reliably with general-purpose equipment and require metrology-grade instrumentation for both signal generation and acquisition. Moreover, the magnetic properties of such small cores and the performance of the sensor component built with them are rarely characterized together, with a complete evaluation of the reference current measurement uncertainty. One of the main contributions of this paper is a specially developed measurement setup based on PXI (PCI eXtensions for Instrumentation) modular instrumentation for the generation and acquisition of the signal. Moreover, the measurement uncertainty is determined in accordance with the GUM and Monte Carlo method. The results for the magnetic properties of the sensor material and current sensor testing are presented in Section 4, and they provide substantial insight into the evaluation of sensor components for DCCT applications.

## 2. DCCT Operating Principle and Theoretical Background

Generally, a DCCT is a magnetic type of current transducer which involves the exploitation of the nonlinear magnetization of ferromagnetic materials for dc current component sensing and a well-known current transformer equivalent model for ac current component sensing [13,14]. Thus, as already mentioned in the Introduction, the dc current component is usually obtained from two dc flux sensing cores which are driven to saturation (with a 180° phase shift) with a modulator circuit. In such a configuration, the third core senses the ac component. If there is no dc flux, the saturation current peaks are equal in both directions. On the contrary, if there is some dc flux, then there is a difference in current peaks proportional to that dc flux. The dc flux mentioned in this case originates from the dc current passing through the current conductor. The conductor is positioned in such a way that it goes through the toroidal core and represents the primary coil (with a number of windings *N* = 1) of the current transformer in that case. Consequently, the common term for the current passing through that conductor is the primary current, which is actually the measured current [15].

**Figure 1:**
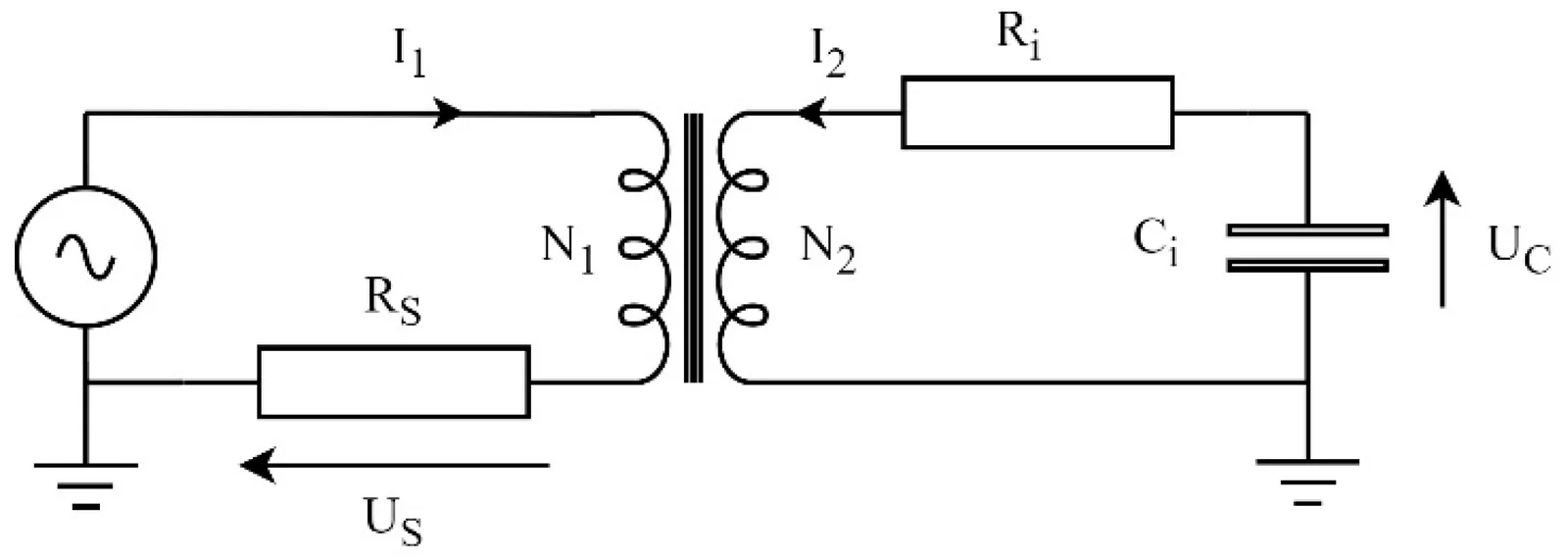
Circuit diagram for the magnetic characterization of toroidal cores.

Regarding the measurement procedure for toroidal core characterization, all measurements are conducted by dynamical hysteresis loop measurements for different maximums of magnetic flux density in a Faraday cage. Saturation flux density and coercive force are determined from the main hysteresis loop. The first magnetization curve is determined by connecting the peak values of Bm, Hm for series of dynamical hysteresis loops in the range from the demagnetized state to the main hysteresis loop. The circuit diagram used in the measurements for the B-H curve and first magnetization curve determination is presented in the Figure 1.

The magnetic field strength is derived from current measurement using the following expression [16]:

(1) $$H(t)=\frac{N_{1}\cdot u_{S}(t)}{l_{m}\cdot R_{S}},$$

where *N*_1_ is the number of turns of the testing winding, and in our measurements, *N*_1_ was set to 100, *u_s_* is the voltage of the current shunt, *l_m_* is the magnetic path length determined based on core dimensions (in all measurements, *l_m_* was set to 0.0413 m) and *R_s_* is the shunt resistance. The values of *R_i_* and *C_i_* were carefully chosen to satisfy the following requirement:

(2) $$R_{i}\gg \frac{1}{\omega \cdot C_{i}},$$

where *R_i_* is the resistance of the *RC* integrator, *C_i_* is the capacitance of the *RC* integrator and *ω* is the angular frequency. Accordingly, the current in the secondary side of the transformer can be approximated by:

(3) $$i_{2}=\frac{e_{2}}{R_{i}},$$

where *e*_2_ is the induced voltage on the secondary side of the transformer. Taking into account that the core specimen has an effective cross-sectional area of *S*, then induced voltage *e*_2_ is defined by:

(4) $$e_{2}=-N_{2}\cdot S\frac{\mathrm{dB}}{\mathrm{dt}}$$

where *N*_2_ is the number of turns of the measuring winding, i.e., of the secondary winding, and *S* is the cross-sectional area of the core with known value of 2.1 × 10^−6^ m^2^. Combining Equations (3) and (4), the current in the transformer secondary is:

(5) $$i_{2}=-\frac{N_{2}\cdot S}{R_{i}}\frac{\mathrm{dB}}{\mathrm{dt}}$$

Generally, a voltage drop across a capacitor is defined by:

(6) $$u_{C}=\frac{1}{C}\int i_{2}\mathrm{dt}$$

It is obvious from Equations (5) and (6) that a voltage drop across a capacitor *u_C_* is proportional to the magnetic flux density *B*. Consequently, the magnetic flux density is derived from the following expression [15]:

(7) $$B(t)=\frac{R_{i}\cdot C_{i}}{N_{2}\cdot S}\cdot u_{C}(t)$$

## 3. Materials and Experimental Methods

**Figure 2:**
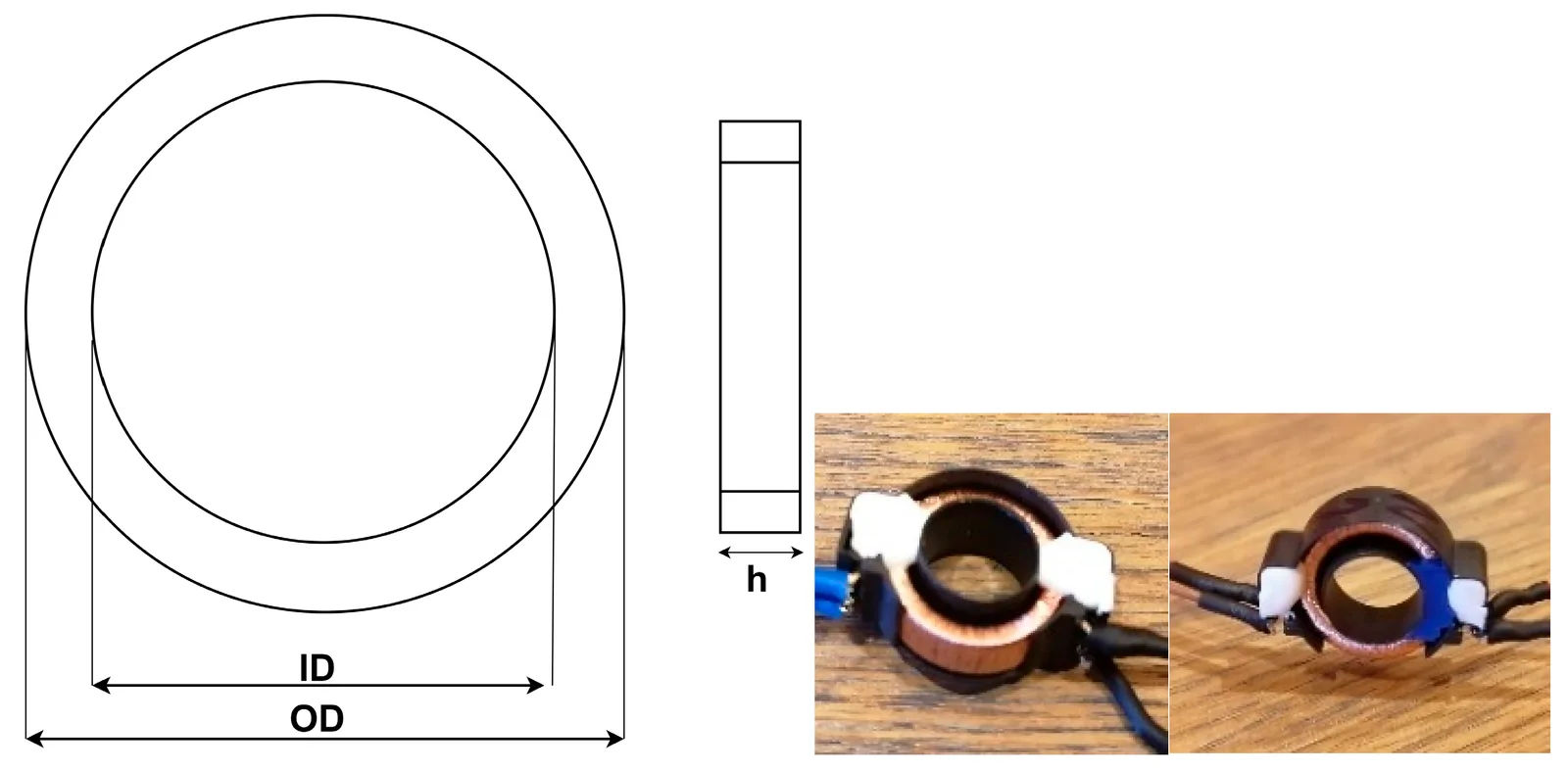
Dimensions of the toroidal cores’ specimens and images of the tested core specimens.

An additional challenge of the measurement was to accurately measure all relevant current and voltage signals primarily due to the very small dimensions of the toroidal core specimens, as presented in Figure 2.

*OD* = 14.2 mm and *ID* = 12.10 mm were the outer and inner dimensions of the toroidal core specimen as marked in Figure 2, while *h* = 2 mm was the height of the toroidal core. The tested nanocrystalline specimens (N1, N2) were custom-made tape-wound toroidal cores produced by VACUUMSCHMELZE GmbH & Co. KG (Hanau, Germany), designed on the basis of the manufacturer’s standard series of epoxy-coated nanocrystalline VITROPERM cores [17]. The cores are made of Fe-based nanocrystalline alloys VITROPERM 500 F. The specimens were taken from regular-series production, and the two specimens of each material were of the same grade and from the same production lot. The nominal composition of VITROPERM 500 F is Fe_73.6_Cu_1.0_Nb_3.0_Si_15.5_B_6.9_ (at.%) [18]. The ribbon is produced by rapid solidification in the amorphous state, wound into toroidal cores and subsequently annealed in a transverse magnetic field to obtain the nanocrystalline structure and a flat hysteresis loop [18]. The tested specimens (P1, P2) were made of a soft ferromagnetic nickel-iron (Ni-Fe) alloy characterized by a high nickel content (typically 75–82 wt.%); this material belongs to the class of high-permeability permalloys [19,20]. The specimens’ characteristics are summarized in Table 1. All cores had the same nominal dimensions, *OD* = 14.2 mm, *ID* = 12.10 mm and *h* = 2 mm, giving a cross-sectional area *S* = 2.1 × 10^−6^ m^2^ and a mean magnetic path length *l_m_* = 0.0413 m, in accordance with IEC 60404-6 [12]. Photographs of the specimens are shown in Figure 2. Thus, metrology-grade instrumentation was used in the measurement setup to satisfy all requirements. The measurement setup’s hardware components were carefully adjusted to ensure that the toroidal core specimens testing and investigation were possible as the specimens’ dimensions were very small and made it challenging to take into account the maximum saturation current they could endure but also to ensure a sufficiently high measurable voltage distinctive from the noise [21]. All measurements were performed as dynamic hysteresis loop measurements for different peak values of magnetic flux density. The toroidal cores were excited at 50 Hz with a sinusoidal magnetizing current, using a magnetizing winding with *N*_1_ = 100 turns and a secondary winding with *N*_2_ = 1000 turns. It consisted of hardware architecture based on a PXIe-1062 modular system with the NI-4461 acquisition module [22,23]. Both the shunt voltage and the induced secondary voltage were sampled simultaneously by an NI 4461 acquisition module in an NI PXIe-1062 modular system, at a sampling frequency of 50 kSa/s. Before each measurement series, the specimens were demagnetized in accordance with IEC 60404-6 and starting from the demagnetized state, the hysteresis loops were then acquired for increasing excitation amplitudes up to the main hysteresis loop. The maximum flux density, remanence and coercivity were determined from the main hysteresis loop, and the first magnetization curve was obtained by connecting the peak values (*H*_m_, *B*_m_) of the series of dynamic hysteresis loops. Each measurement was repeated three times. Moreover, the NI-4461 module has two output channels. Analog output signals from these channels were used as excitation signals and forwarded to a Toellner TOE 7621 power amplifier [24]. A precise cage ac current shunt with a nominal current of 300 mA (FER ZOEEM SN0003) and a nominal resistance of 2.14 Ω was used for precise current measurement on the primary side [25]. As already mentioned, the RC integrator consisted of *R_i_* and *C_i_*, where *R_i_* = (382.93 ± 0.27) kΩ, *C_i_* = (2.9752 ± 0.0176) µF. All measurements were performed in a laboratory with a controlled room temperature of (23 ± 1) °C.

**Figure 3:**
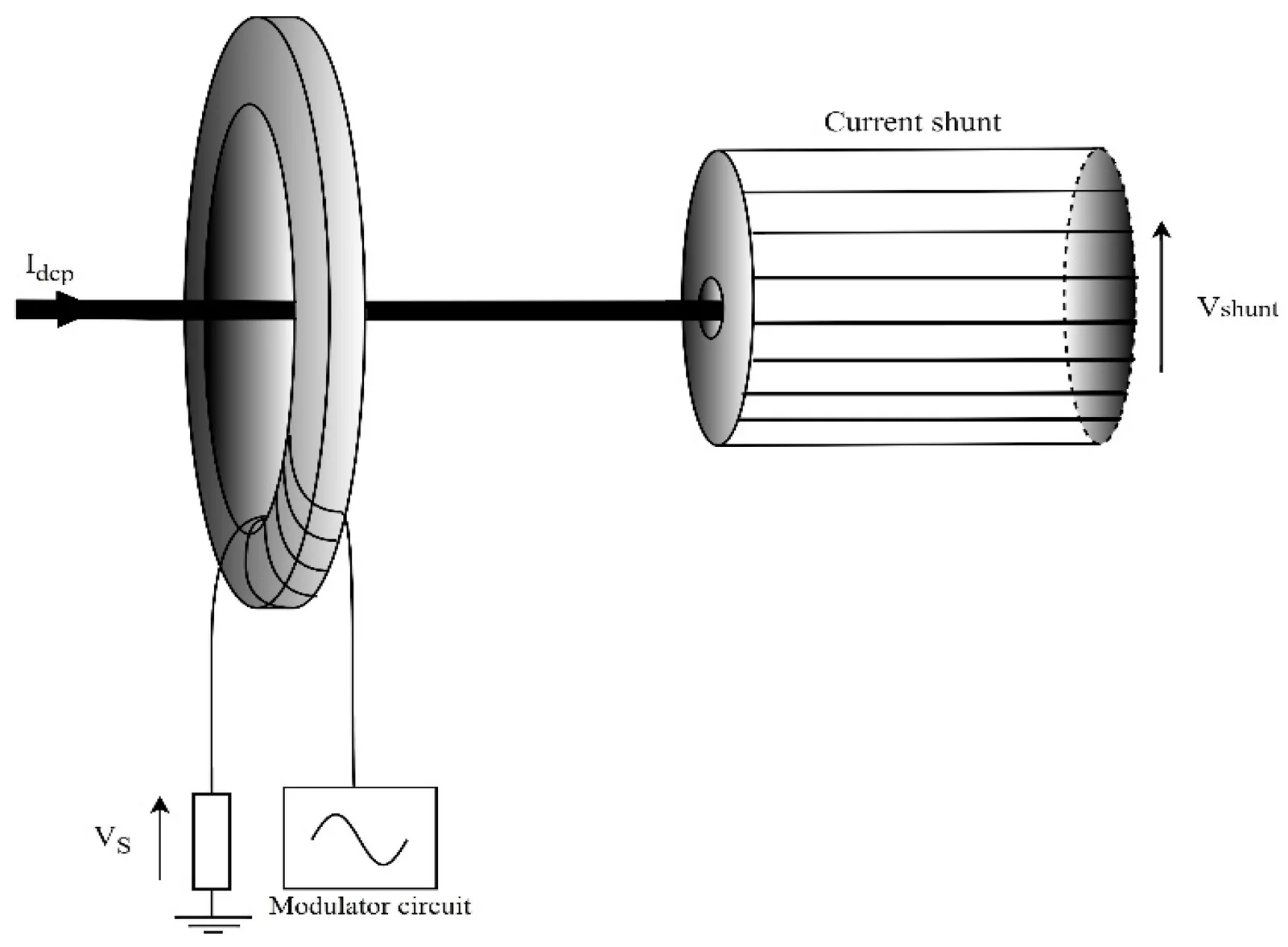
Test measurement setup scheme for DCCT sensor component.

The second developed test measurement setup consisted of metrology-grade components to determine the overall linearity of the tested sensor. A modulator circuit consisted of two components, i.e., a function generator and a Toellner TOE 7621 power amplifier. It provided an excitation current to drive the core to saturation. The sensitivity of the investigated sensors for DCCT applications can be improved by applying a higher excitation current frequency. However, a tradeoff is required since a high excitation current frequency increases eddy currents in the core material [2]. Taking that into account and to achieve low power consumption in the future designing stage of DCCT according to [11], the excitation frequency was selected to be 500 Hz. Accordingly, in this measurement setup, a winding with a higher number of turns, i.e., with *N* = 1000, was utilized for driving the core to saturation. The measurement setup scheme for testing the DCCT sensor component in the configuration with one toroidal core is presented in Figure 3.

Regarding the signal extraction and calibration, the measured dc current was provided by a DP1116A programmable DC power supply [26]. Further, that dc current was applied to the cage current shunt (FER ZOEEM SN0006) with a nominal current of 10 A and nominal resistance of 71.4 mΩ [27,28]. The output voltage of the cage current shunt *V_shunt_* was then sampled by one channel of the NI 4461 digitizer, representing the reference dc current readings [22]. The toroidal core was driven to saturation with the already mentioned modulator circuit. The saturation current passed through the resistor, which in our case was also the cage current shunt a with nominal resistance of 35.7 Ω (FER ZOEEM SN0007), and the voltage at its output was denoted V_S_. The output voltage *V_S_* was then sampled by the other channel of the NI 4461 digitizer. When the measured dc current was applied, it created a dc flux and caused a difference in current peaks which were proportional to that dc flux.

The sensor output was derived from the voltage *V*_S_ across the sampling resistor *R*_S_ = 35.7 Ω connected in series with the 1000-turn winding (channel ai0), while the reference current was obtained from the voltage across the reference shunt FER ZOEEM SN0006 (*R*_shunt_ = 71.4118 mΩ, channel ai1), *I*_ref_ = *V*_shunt_/*R*_shunt_ (Equation (9)). The core was excited at 500 Hz (580 mV_pp_ at the function generator, amplified by a TOE 7621 amplifier in CV mode with a 1 kHz bandwidth). Both channels of the NI 4461 (pseudo-differential mode) were sampled at 200 kS/s, and each record contained 4000 samples (20 ms, i.e., 10 excitation periods). In each record, the positive and negative peak values of *V*_S_ were determined as signed quantities, *V*_p+_ = max *V*_S_(*t*) and *V*_p−_ = min *V*_S_(*t*), and the sensor output was defined as

(8) $$V_{\mathrm{out}}=\frac{(V_{p+}+V_{p-})\;}{2}$$

Because signed peaks were used, *V*_out_ was zero for symmetrical peaks (*I*_dcp_ = 0) and changed sign with the polarity of *I*_dcp_. Each calibration point was the mean of 10 consecutive records. The sensor was calibrated for reference currents from −4 A to +4 A, covering both polarities. A linear least-squares fit gave the calibration equation

*V_out_* = *S* · *I _ref_* + *b* (9)

with a sensitivity *S* = 28.60 mV/A and an intercept *b* = 0.56 mV (*R*^2^ = 0.99997). The measured current was obtained by inverting Equation (10), *I*_dcp_ = (*V*_out_ − *b*)/*S*, and the linearity error was related to the full scale *I*_FS_ = 4 A:

(10) $$\varepsilon =\frac{\left(I_{\mathrm{dcp}}-I_{\mathrm{ref}}\right)}{I_{\mathrm{FS}}}\cdot 100\%$$

## 4. Results and Discussion

**Figure 4:**
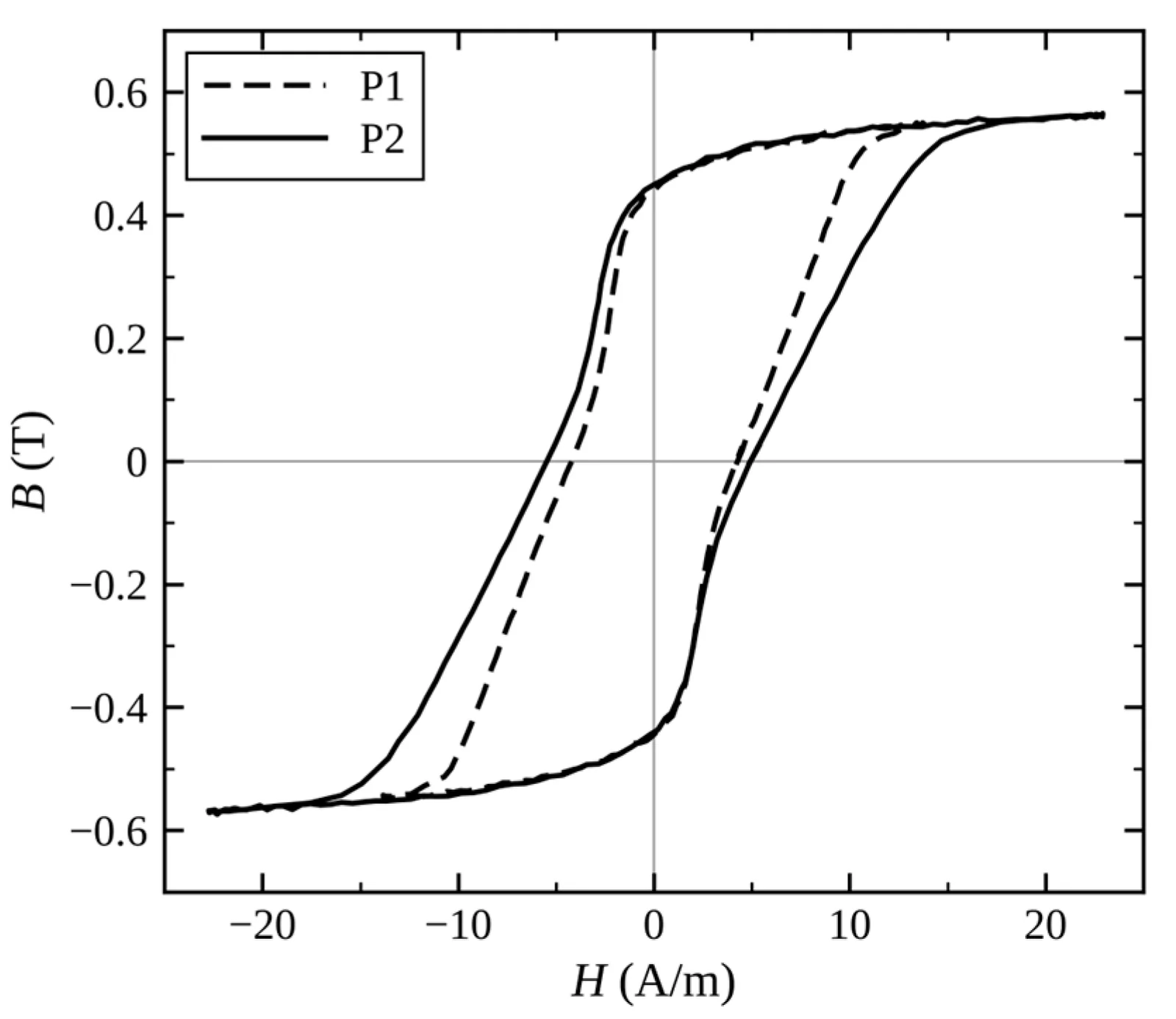
B-H curves of two permalloy toroidal core specimens.

**Figure 5:**
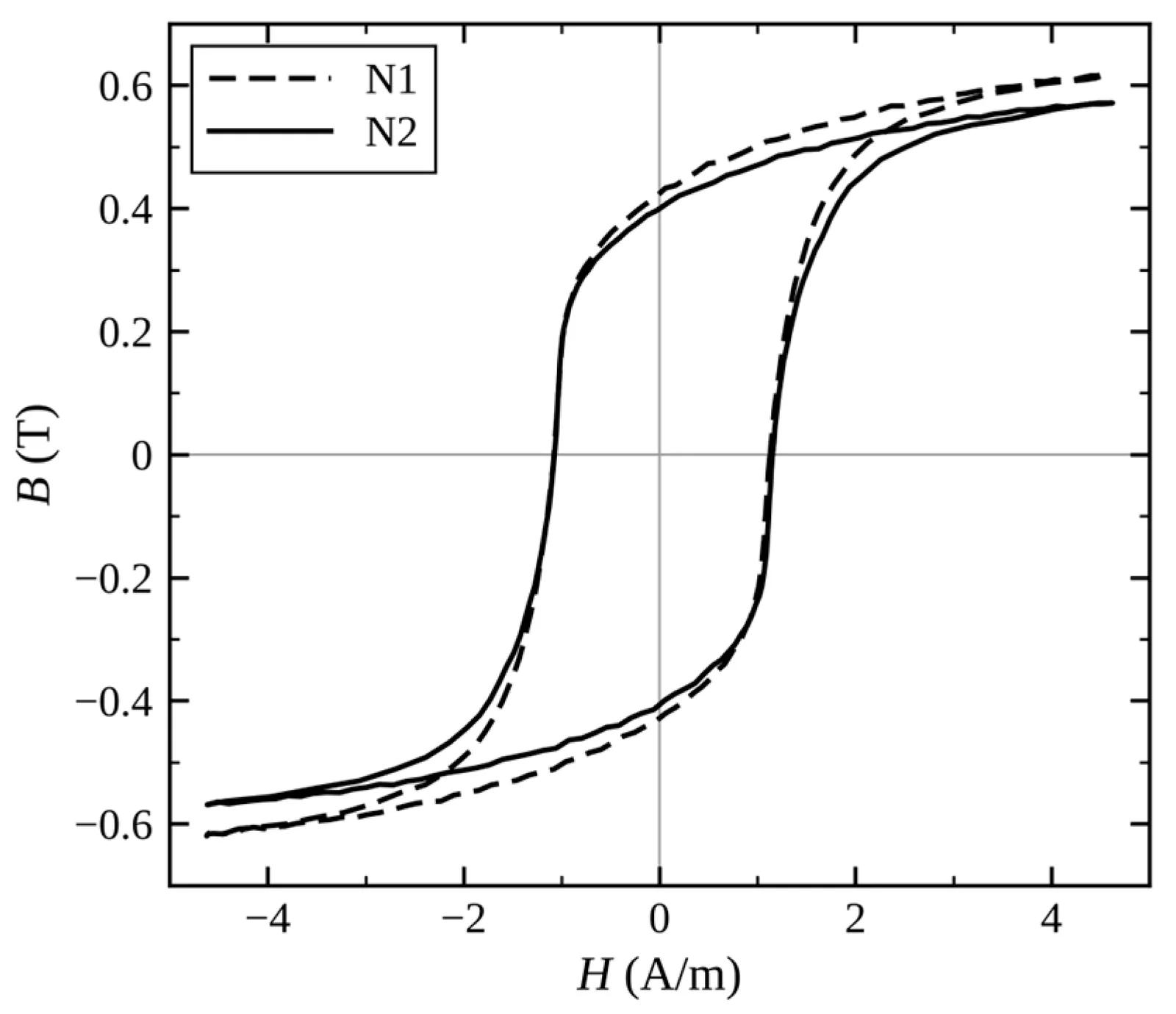
B-H curves of two nanocrystalline toroidal core specimens.

Results obtained with the measurement method described in Section 2 are presented in the following figures. The whole measurement procedure was programmed and automatized using the LabVIEW programming environment. Measurement samples acquired with the NI 4461 were further processed to calculate parameters of interest. Specifically, to obtain the B-H curve, Equations (1) and (7) were used. The B-H curves are presented for two different permalloy toroidal core specimens in Figure 4 and for two different nanocrystalline toroidal core specimens in Figure 5.

As can be seen, the B-H curves of nanocrystalline toroidal core specimens are practically overlapping which is a favorable property with regards to load symmetry in the modulation stage. On the other hand, the B-H curves of permalloy toroidal core specimens exhibit slight deviations in the first and third quadrant on the respected coordinates. Obviously, all investigated cores offer extremely low coercivity *H_c_*. Accordingly, the measured *H_c_* of nanocrystalline cores is 1 A/m, while the *H_c_* of permalloy cores is around 4 A/m. Both nanocrystalline and permalloy cores have saturation flux density Bs at around 0.6 T. Taking all mentioned measurement results into account, it can be concluded that both materials are good fit in DCCT sensor applications. Thus, both materials exhibit high permeability in linear mode and low driving current. The first magnetization curves are presented for both materials in Figures 8 and 9. Those curves were determined by recording commutation points only, i.e., by recording the peak values *H_m_* and *B_m_* at each step. Once those results were obtained, it was possible to calculate the relative magnetic permeability *µ_r_*:

(11) $${µ}_{r}(B)\;=\;\frac{B_{m}}{{µ}_{0}H_{m}}$$

**Figure 6:**
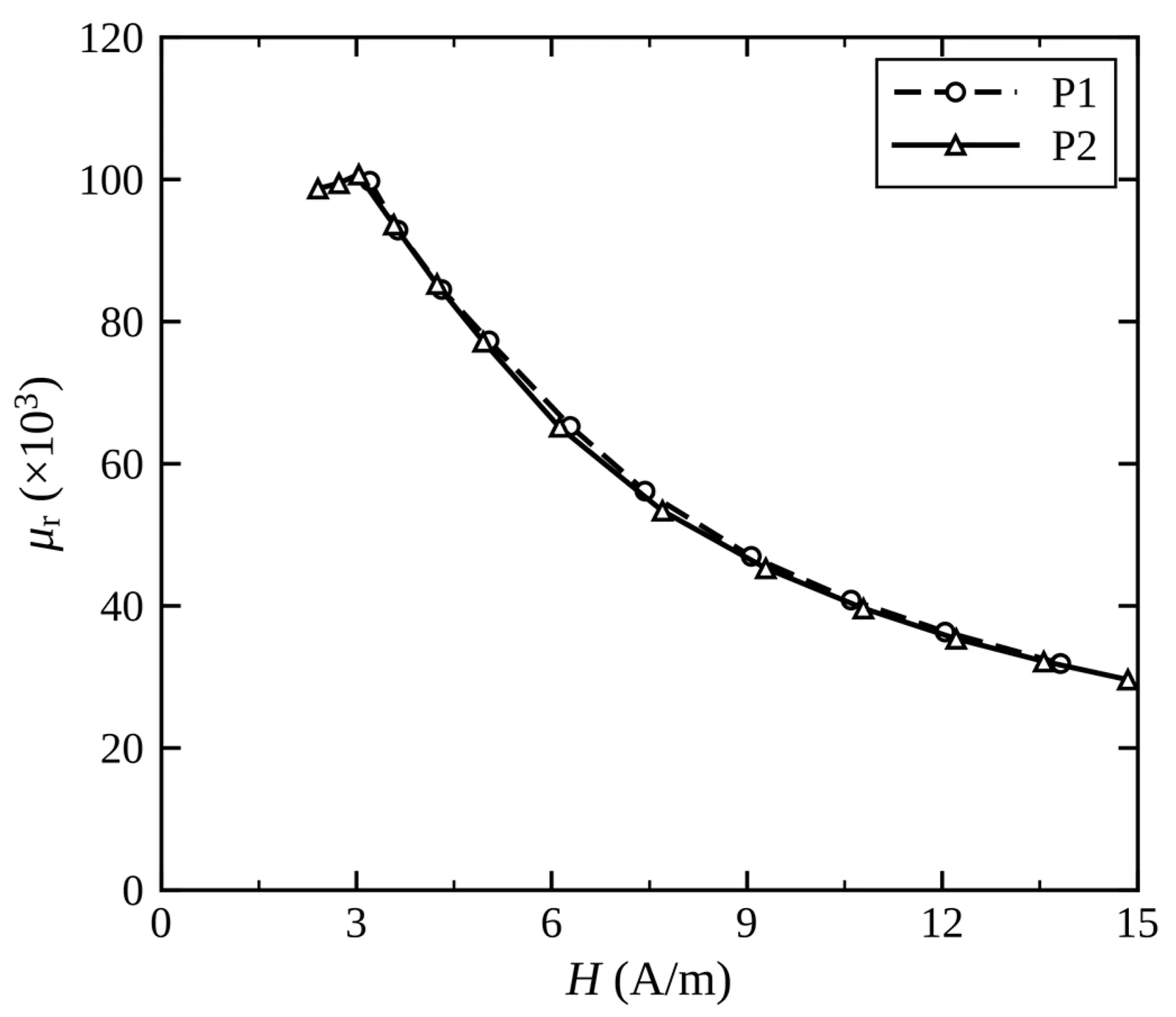
Relative magnetic permeability of two permalloy toroidal core specimens.

**Figure 7:**
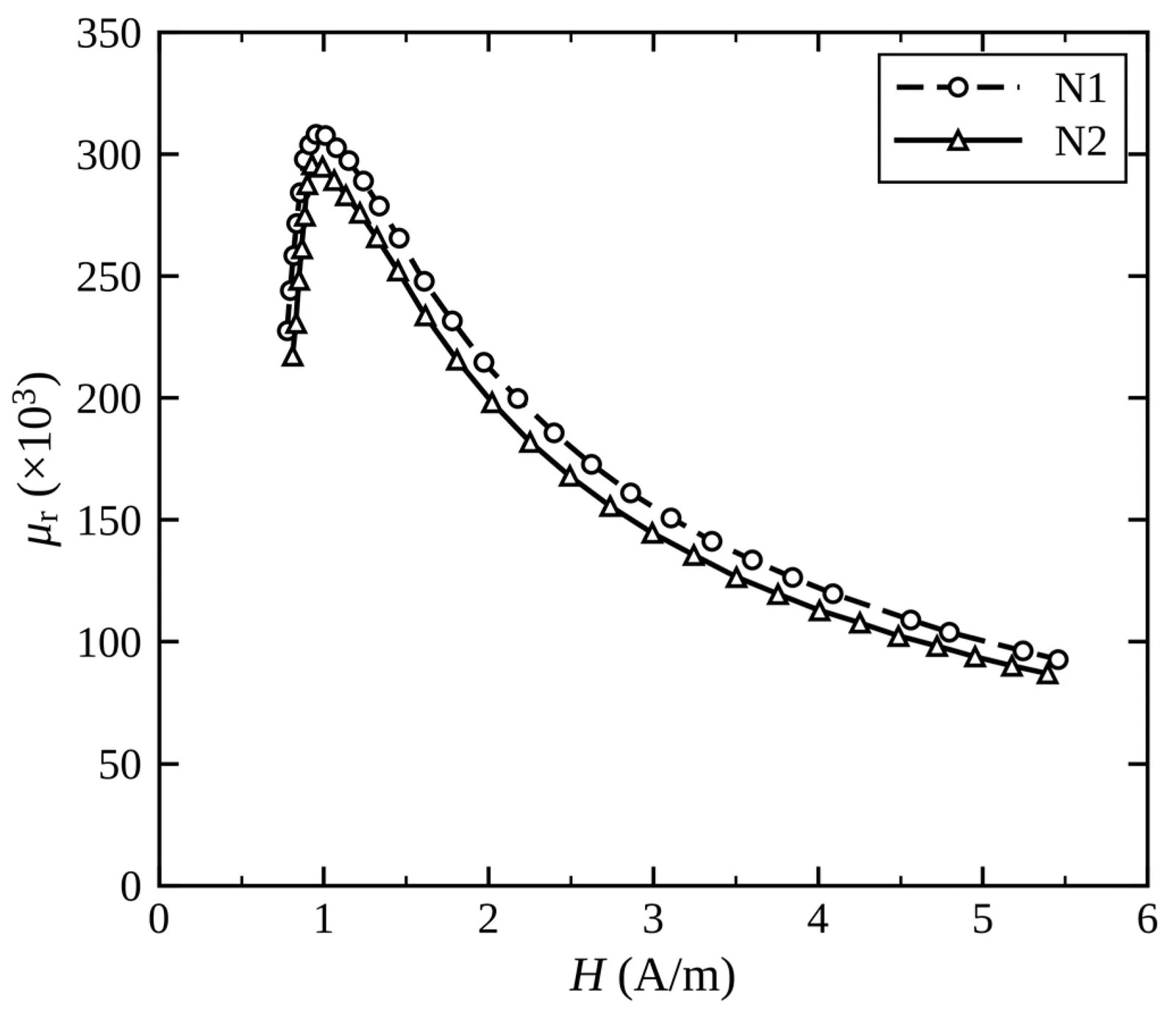
Relative magnetic permeability of two nanocrystalline toroidal core specimens.

According to (11) and as can be seen from the following graphs, the relative magnetic permeability *µ_r_* of nanocrystalline cores goes up to 3 × 10^5^, while the relative magnetic permeability of permalloy cores goes up to 1 × 10^5^. The relative magnetic permeability results are represented as a function of the magnetic field intensity *H* in Figure 6 and Figure 7.

Although both materials showed favorable magnetic properties for DCCT sensor application, the nanocrystalline core specimens tested in this study exhibited slightly more suitable magnetic properties for DCCT and as such were further investigated for the sensor component, as detailed in the following sections. Thus, a lower *H_c_* and higher relative magnetic permeability *µ_r_* in linear mode in the end facilitates the thermal management and choice of all other electronic components in the potential future design stage of DCCTs [3,4].

**Figure 8:**
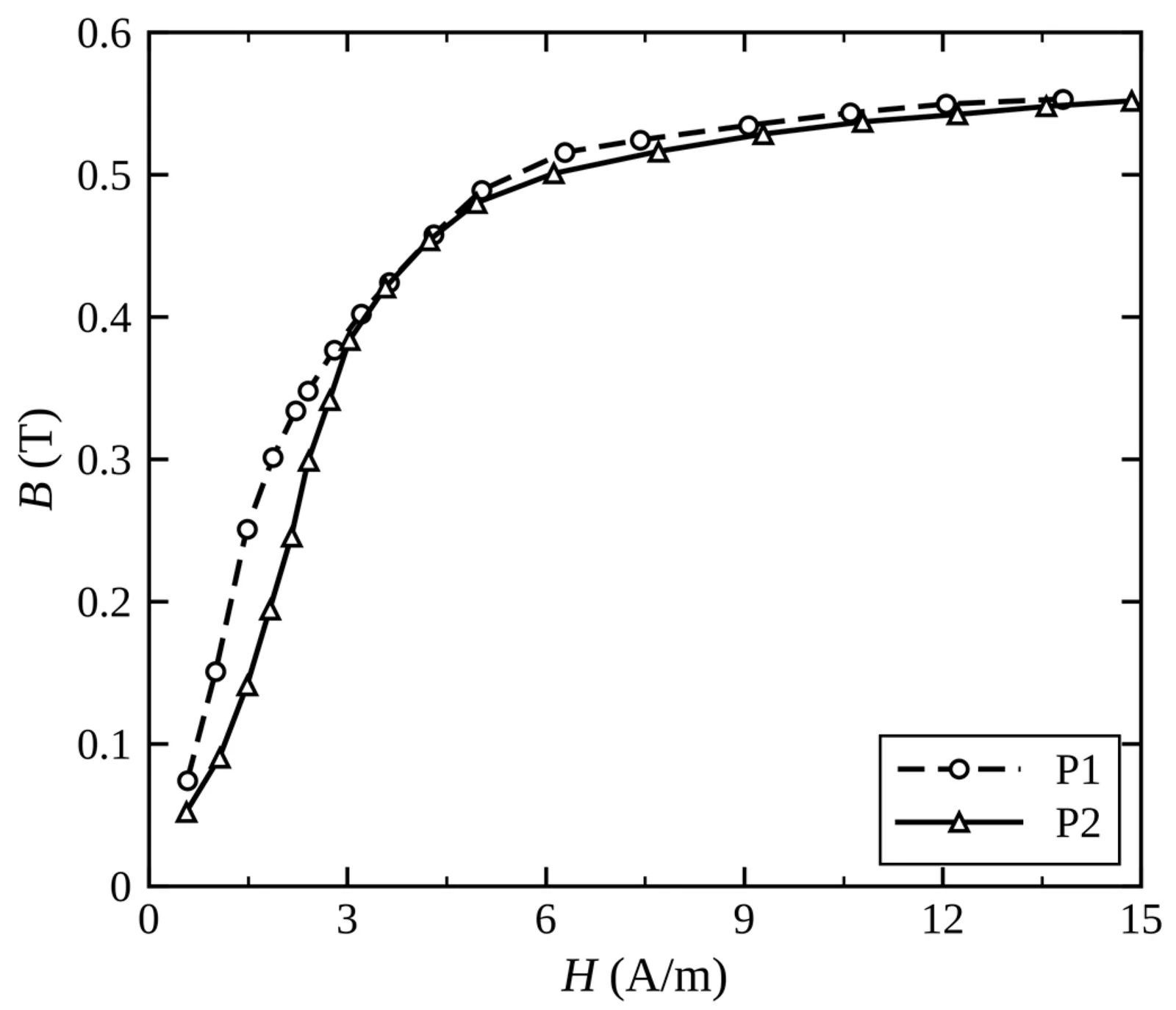
First magnetization curves of two permalloy toroidal core specimens.

**Figure 9:**
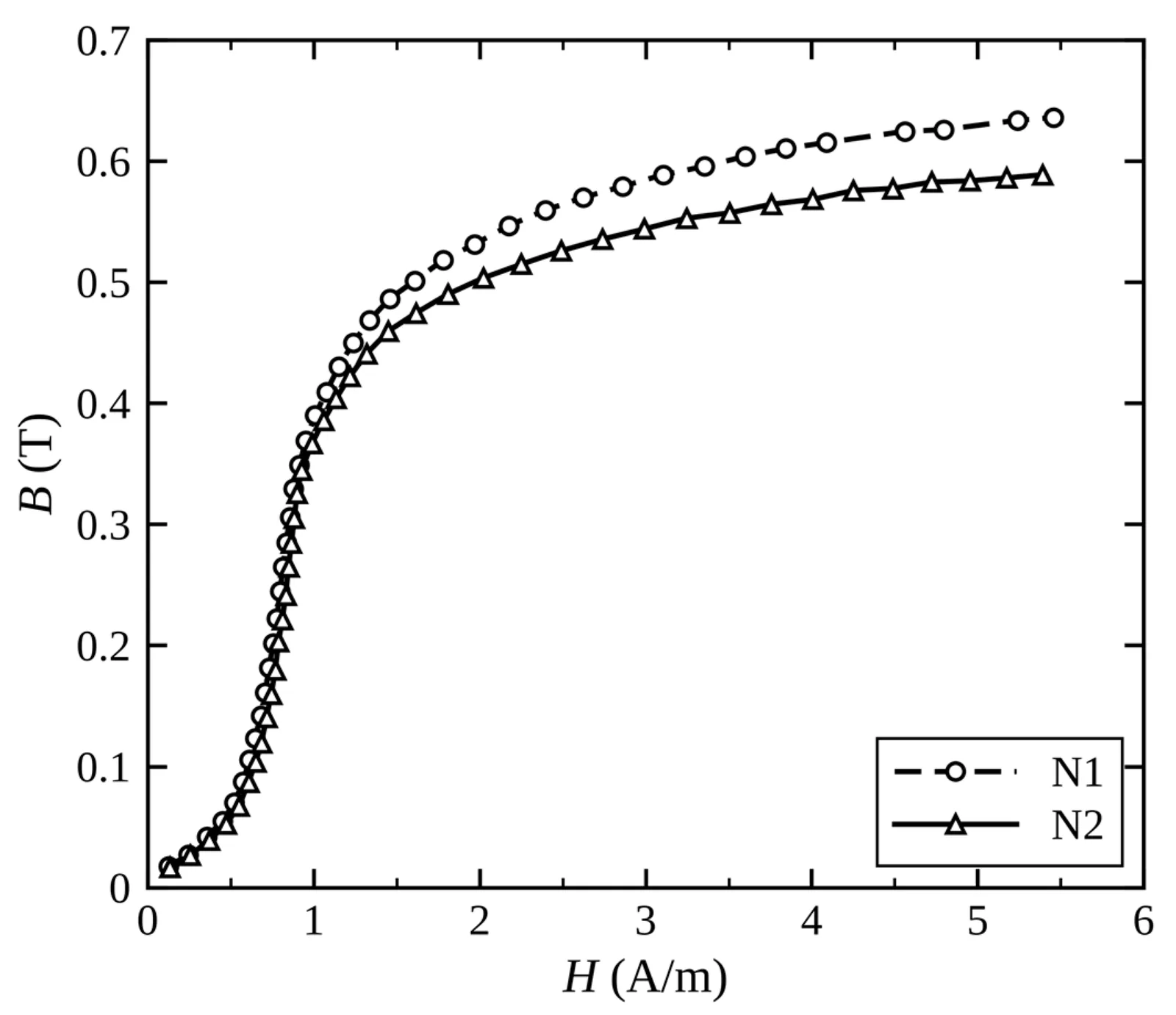
First magnetization curves of two nanocrystalline toroidal core specimens.

The measured magnetic properties of the tested specimens, determined from the measured data shown in Figure 4, Figure 5, Figure 6, Figure 7, Figure 8 and Figure 9, are summarized in Table 1. All uncertainties are expanded uncertainties for a coverage probability of approximately 95%. The measurement uncertainty was evaluated in accordance with the GUM [29], combining the Type B contributions of the digitizer and shunt resistor with the Type A contribution of three repeated measurements.

**Table 1:** Numerical summary of the measured properties with uncertainties.

Specimen	*B*_max_ (T)	*H*_max_ (A/m)	*B*_r_ (T)	*H*_c_ (A/m)	*μ*_r,max (×10_^3^_)_	*H*(*μ*_r,max_) (A/m)
P1	0.55 ± 0.03	13.8 ± 0.6	0.44 ± 0.02	4.24 ± 0.19	100 ± 5	3.21
P2	0.57 ± 0.03	14.9 ± 0.7	0.45 ± 0.02	4.88 ± 0.29	101 ± 5	3.03
N1	0.62 ± 0.03	4.59 ± 0.21	0.43 ± 0.02	1.10 ± 0.04	308 ± 17	0.95
N2	0.57 ± 0.03	4.62 ± 0.21	0.40 ± 0.02	1.10 ± 0.12	296 ± 16	0.93

The results in Table 1 quantify the differences between the tested materials. Nanocrystalline specimens N1 and N2 reached a maximum relative permeability of approximately 300 × 10^3^, about three times higher than permalloy specimens P1 and P2 (approximately 100 × 10^3^), and a coercivity of approximately 1.1 A/m, about four to five times lower than that of the permalloy specimens (4.2 to 4.9 A/m). Moreover, the nanocrystalline specimens reached a flux density of 0.57–0.62 T at a peak field strength of only about 4.6 A/m, whereas the permalloy specimens required about 14 A/m to reach 0.55 T. For a sensor that relies on periodic saturation of the core, this means that the nanocrystalline cores require an excitation field about three times lower, and thus a lower excitation current and power, and their lower coercivity reduces the hysteresis losses and the offset associated with remanence. The comparison in Table 1 is based on two specimens of each material of the same grade and from the same production lot. Such a sample set does not allow a statistical evaluation of the production spread, and the results cannot be generalized to other production lots, grades, manufacturers or core geometries.

**Figure 10:**
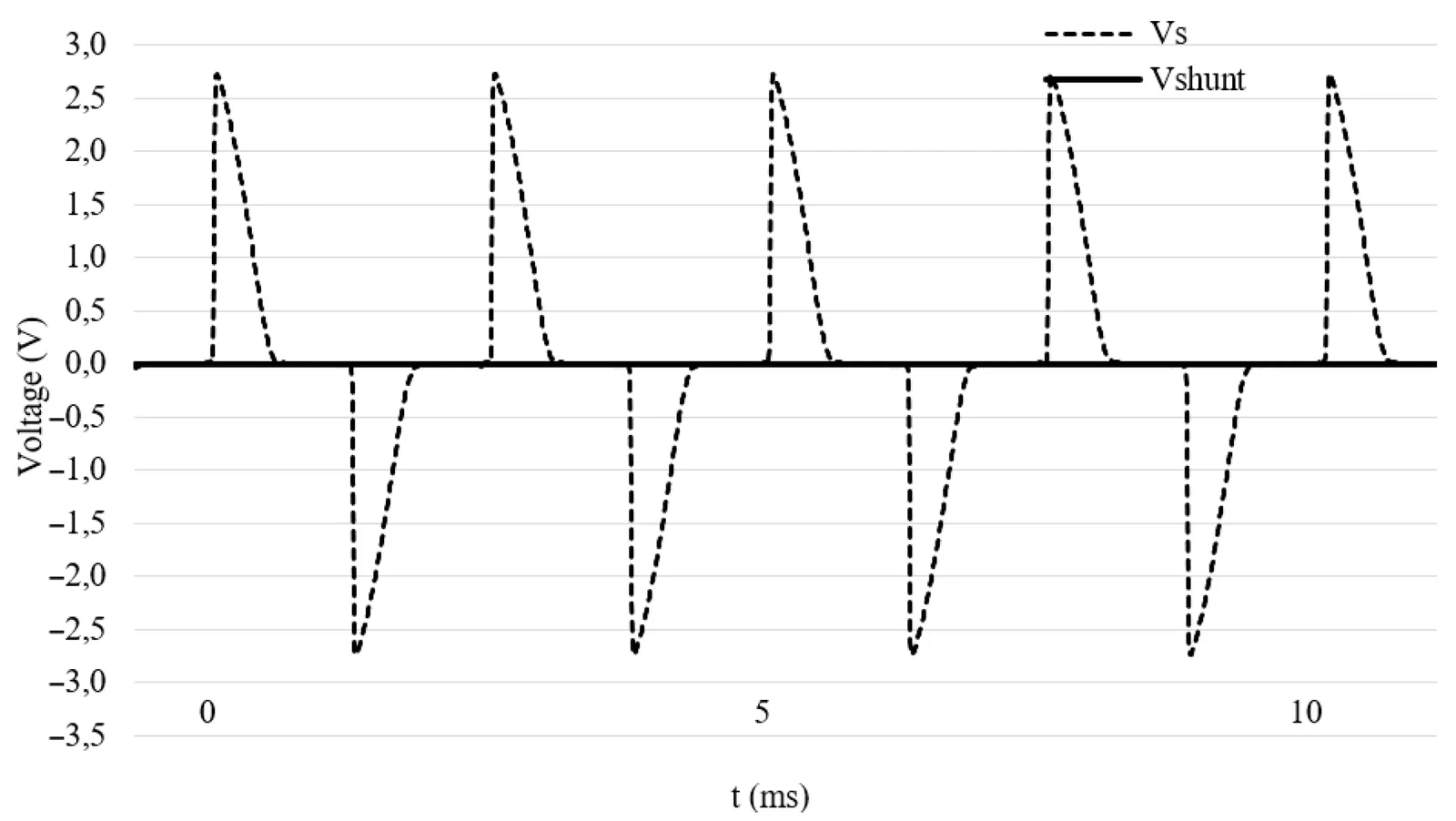
Acquired voltage waveforms when *I_dcp_* = 0 A.

The following results were obtained using the measurement setup described in the previous section and depicted in Figure 10. The relevant signals were acquired with the NI 4461, and the relevant samples were processed to calculate the current peak difference obtained with the investigated sensor and the reference current readings obtained with the current shunt. The reconstructed voltage waveform *V_S_* of the sampling resistor *R_s_* and *V_shunt_* of the current shunt are shown in Figure 10 for a measured or primary current *I_dcp_* of 0 A.

**Figure 11:**
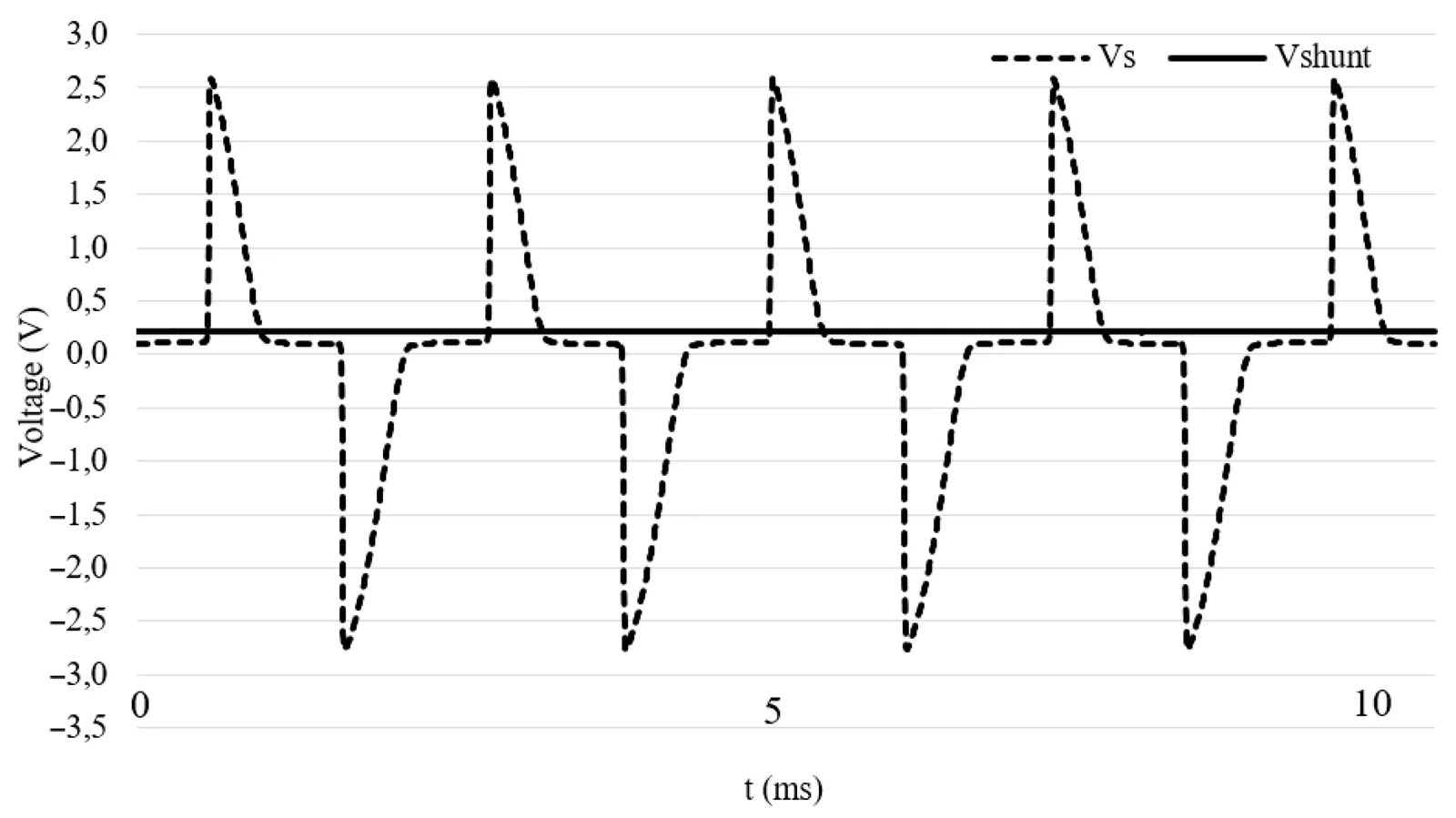
Voltage waveform when *I_dcp_* = 3 A.

**Figure 12:**
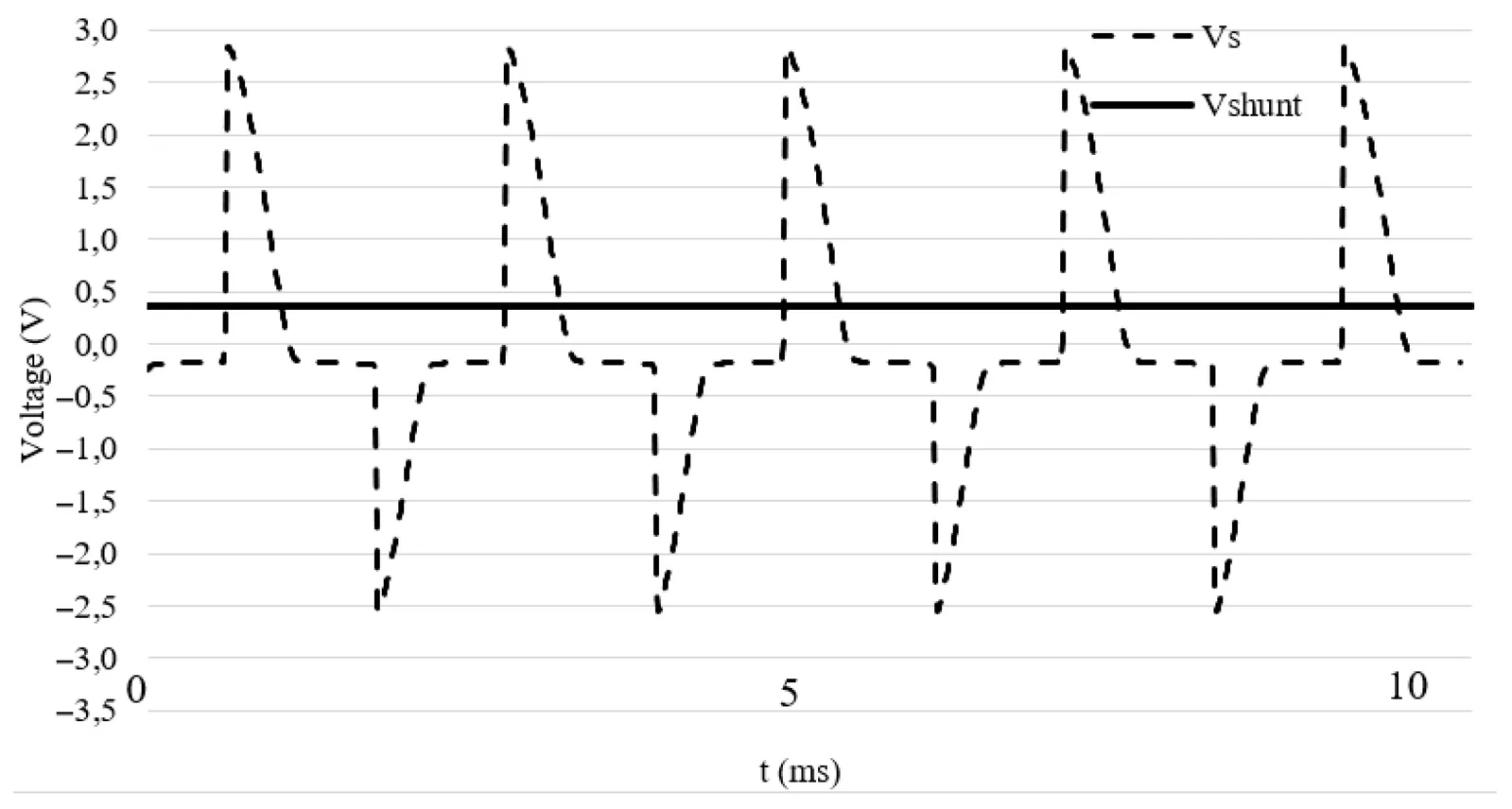
Voltage waveform when *I_dcp_* = 5 A.

When there is some *I_dcp_*, then the created dc flux is visible as an asymmetry in the current peaks (i.e., the peaks of signal *V_S_*) as shown in Figure 11 and Figure 12.

As the applied current rises, *V_shunt_* also rises as is obvious from the presented graphs. In accordance with that, the average values of *V_shunt_* and related standard deviations are presented in Table 2.

Obviously, the stability of the output voltage from the referent cage current shunt is around 160 µV. The value of −23 × 10^−6^ V obtained for *I*_dcp_ = 0 A represents the zero offset of the reference measurement chain (offset of the digitizer channel and thermoelectric voltages at the shunt terminals). It corresponds to −0.3 mA, which is well below the standard deviation of the record and therefore not significant. The standard deviation of approximately 160 µV (about 2.2–2.4 mA) is practically independent of the applied current; it is mainly caused by the 500 Hz component induced from the excitation winding into the single-turn primary conductor and superimposed on the measured dc current.

**Figure 13:**
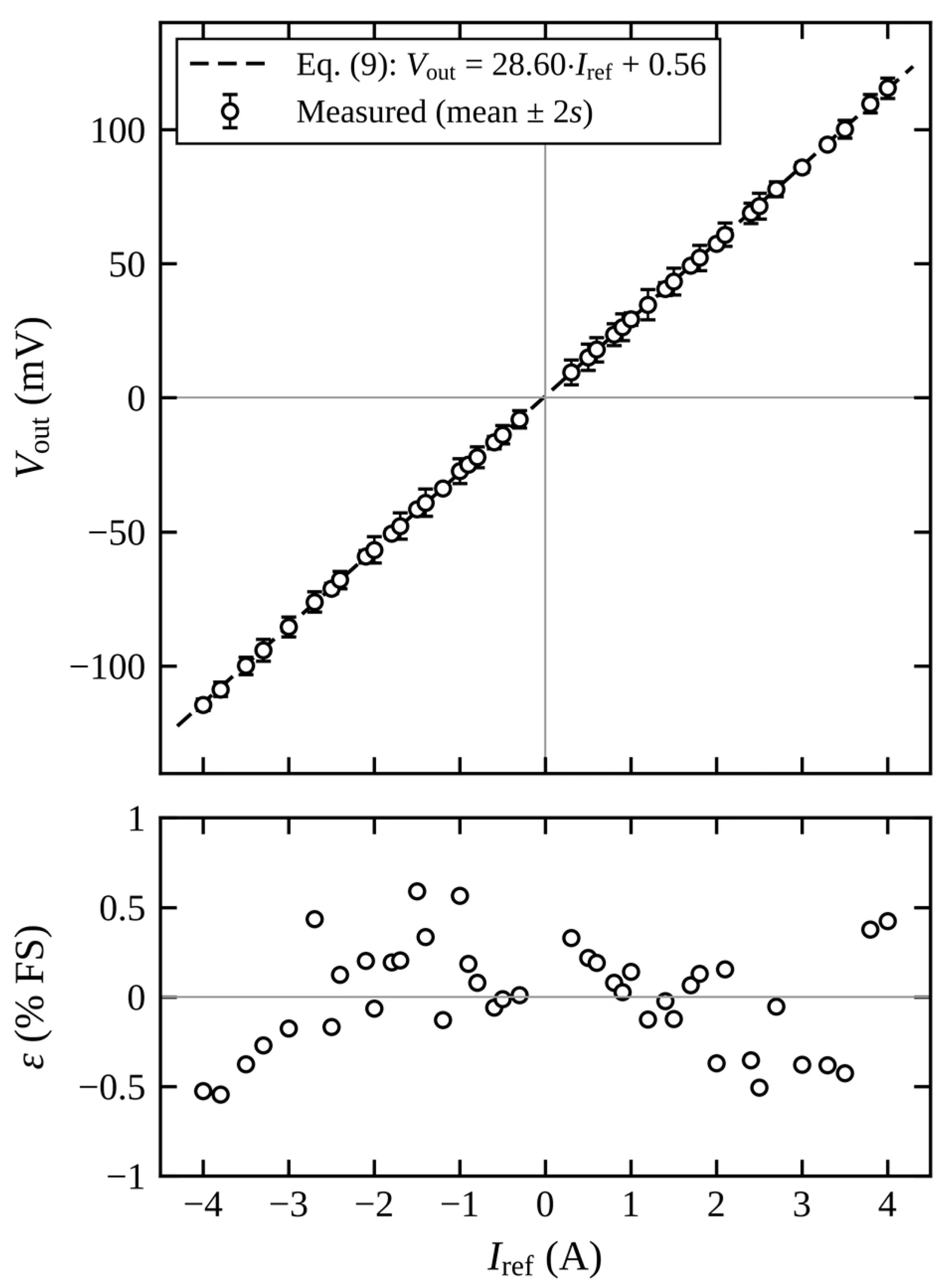
Calibration of the sensor: output *V*_out_ versus reference current *I*_ref_ for both polarities (42 calibration points; error bars: ±2s of 10 records), with the linear fit of Equation (9) (**top**), and the linearity error *ε* (**bottom**).

The calibration plot is shown in Figure 13. At each calibration point, the mean sensor output of the 10 records was used; the calibration equation was obtained by an unweighted linear least-squares fit of these mean values against the reference current, covering both polarities in a single fit. The linearity error was calculated as a signed quantity for each calibration point, and the signed errors, shown in the lower part of Figure 13, range from −0.55% to +0.59% FS. The reported maximum linearity error of 0.59% FS is the maximum absolute value of these errors. The standard deviation of *V*_out_ within the 10 records of each calibration point (in total 42 calibration points or 21 for each polarity) ranges from 0.3 mV to 2.8 mV, which corresponds to 10 mA to 98 mA for a single record; these standard deviations were used only to characterize the repeatability (error bars in Figure 12) and not as weights in the fit. The residuals of the fit are of the same order as the repeatability and are randomly distributed for both polarities, with mean values of −0.03% FS for the positive and +0.03% FS for the negative calibration points. Hysteresis was not evaluated separately: the calibration points of each polarity were measured once, in a single sweep, e.g., from 0.3 A to 4 A for the positive one. Above approximately 4 A, the sensor response became nonlinear, which defined the upper limit of the measurement range for the tested core specimens.

Current sensors based on magnetic cores are commonly realized either in an open-loop or in a closed-loop (compensated, zero-flux) configuration [30]. Open-loop sensors are simpler, smaller, cheaper and require less supply power, but their linearity and gain accuracy are moderate, and their dc offset drifts with temperature. In closed-loop sensors, negative feedback considerably improves the linearity and temperature stability, at the cost of a more complex construction, larger size, higher cost and higher power consumption [30]. The maximum linearity error of 0.59% FS obtained in this work refers to the open-loop characteristic of the sensor component, obtained with simple signed-peak detection over a range of ±4 A. This is of the same order as the relative linearity error of 0.25% reported for a low-power self-oscillating fluxgate current sensor [30]. Also, linearity results presented here are lower than a fluxgate current sensor based on magnetization residence times, whereas a linearity error of 2.17% over a 6 A range has been reported with the traditional residence-time-difference method [29]. However, this linearity error can be improved by neural-network-enhanced signal processing to 0.054% over a 15 A range, as reported in [31]. Regarding the closed-loop configuration, linearity errors have been reported to be around 0.11% in [11].

The linearity of the tested sensor component is therefore better than the traditional signal processing mentioned in [31] but lower than that of sensors with optimized signal processing or magnetic circuits. Such results are expected, as the core was tested in the open-loop configuration, and the linearity of a complete DCCT is expected to be considerably better when the sensor component operates as a null detector in a zero-flux configuration, in which its nonlinearity is largely suppressed by the feedback, as explained in [30]. Since the feedback keeps the core close to the zero-flux state, the measurement range is no longer limited by the onset of core saturation, so a wider measurement range can also be achieved.

## 5. Uncertainty Analysis of the Reference Current Measurement Using GUM and Monte Carlo Method

The verification of the proposed setup was made by analyzing the amplitude contributions. Both acquired signals were converted into voltage with current transducers, i.e., cage current shunts. Signals were then acquired with the NI 4461, and the relevant samples were processed to calculate the current peak difference obtained with the investigated sensor and reference current readings obtained with the shunt. The measurements were conducted under nominal laboratory conditions. The air temperature was kept constant within the limits of (23.0 ± 1.0) °C, with a relative humidity of (50 ± 10)%. The current shunts utilized in this paper have been thoroughly characterized by their dc and ac properties in various research papers [25,27]. Based on these references, the contributions of the reference shunt (calibration of the dc resistance, drift, and the effects of temperature, power and humidity) were estimated conservatively and expressed as absolute standard uncertainties of the shunt resistance (Table 3). The amplitude uncertainty of the NI 4461 digitizer was taken from [28]. The reference current was obtained from the model in the form of the following equation [32,33]:

(12) $$I=\frac{V_{\mathrm{shunt}}\;\cdot \;(1+\;\delta_{S}\;+{\;\delta }_{\mathrm{dig}})}{R_{\mathrm{shunt}}\;\cdot \;(1+\;\delta_{\mathrm{Rs}}\;+\;\delta_{\mathrm{drift}\;}+\;\delta_{\mathrm{ac}-\mathrm{dc}}\;+{\;\delta }_{\mathrm{TCR}}\;+\;\delta_{\mathrm{PCR}}+\;\delta_{\mathrm{RH}})},\;$$

where *V_shunt_* is the measured output voltage of the current shunt, and *R_shunt_* is the dc resistance of the current shunt. The coefficient in the numerator *δ_S_* is a correction due to the repeatability of the ac voltage measurement. Further, *δ_dig_* is a correction due to the digitizer deviation, and both corrections are assumed to be zero within uncertainty *u(δ_S_), u(δ_dig_),* respectively, with a normal distribution. The coefficient in the denominator *δ_Rs_* is a correction due to the dc resistance measurement deviation, *δ_drift_*, due to the drift of the shunt, *δ_ac-dc_*, due to the ac–dc difference of the shunt, *δ_TCR,_ δ_PCR_* and *δ_RH_* are corrections due to the temperature, power and relative humidity coefficient, respectively [25,27]. The sensitivity coefficients were obtained by partial differentiation of Equation (12):

(13) $$c_{V}=\frac{\partial I_{\mathrm{ref}}}{\partial V_{\mathrm{shunt}}}=\frac{1}{R_{\mathrm{shunt}}}=14.003\;A/V,c_{R}=\frac{\partial I_{\mathrm{ref}}}{\partial R_{\mathrm{shunt}}}=-\frac{V_{\mathrm{shunt}}}{{R_{\mathrm{shunt}}}^{2}}=-70.030\;A/\Omega$$

All input quantities originated from independent sources and were treated as uncorrelated, so the combined standard uncertainty was

(14) $$u_{c}\left(I_{\mathrm{ref}}\right)=\sqrt{{\left(c_{V}\cdot u(V_{\mathrm{shunt}})\right)}^{2}+{\left(c_{R}\cdot u(R_{\mathrm{shunt}})\right)}^{2}}$$

with *u*(*V*_shunt_) = 165.3 µV and *u*(*R*_shunt_) = 1.13 µΩ (15.9 µΩ/Ω) obtained as the root sum of squares of their components (Table 3). The repeatability of *V*_shunt_ was evaluated conservatively as the standard deviation of the individual samples within a record. Normalized to 1 A of the measured current, i.e., in relative terms with sensitivity coefficients of +1 for *V*_shunt_ and −1 for *R*_shunt_, this corresponds to 463 µA/A and an expanded uncertainty of ± 926 µA/A (*k* = 2), as summarized in Table 3. The uncertainty is dominated by the repeatability of the voltage measurement and by the digitizer; the contributions of the shunt are below 0.2% of the combined variance.

**Figure 14:**
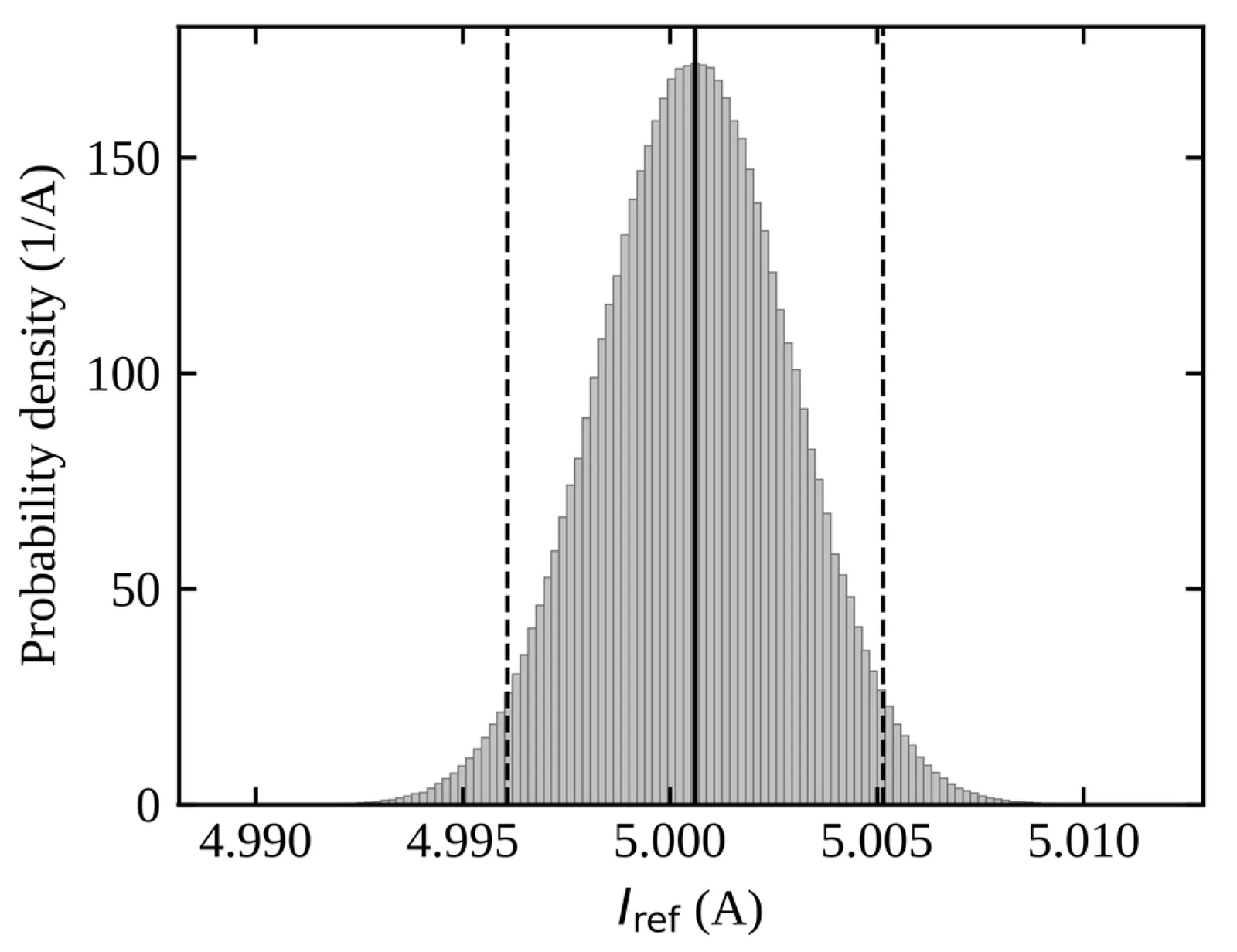
Current distribution estimated from 10,000 MC runs.

Another measurement uncertainty analysis was carried out using the Monte Carlo (MC) method implemented in Python 3.14.6. programming language, using exactly the same input quantities and standard uncertainties as in Table 3. In each of 10,000 trials, a value of each input quantity was drawn from its assigned distribution (normal for the Type A and calibration contributions, rectangular for the drift, temperature, power and humidity effects) and propagated through the full model of Equation (12). The resulting distribution of the reference current is shown in Figure 14, with its 95% coverage interval of ±906 µA/A. The endpoints of the GUM and Monte Carlo intervals differ by 20 µA/A.

## 6. Conclusions

In this paper, we proposed a realization of two specially developed measurement setups consisting of metrology-grade pieces of equipment. Industry requirements are often set to conduct a number of control measurements to check magnetic properties of the ordered sensor material. Accordingly, it often requires application of existing measurement methods but with specific constraints. Based on the presented results, the contribution of this paper lies in the application and integration of established measurement methods, namely, dynamic hysteresis loop measurements on ring specimens and the detection of dc current from the asymmetry of the saturation current peaks, into a laboratory test setup for the characterization of DCCT sensor components. Using that setup, the magnetic properties of the tested permalloy and nanocrystalline core specimens were determined with stated uncertainties. The sensor component was calibrated for both current polarities, with a linearity error of 0.59% FS in the range from −4 A to +4 A. A further contribution is the evaluation of the measurement uncertainty of the reference current measurement in the proposed setup, carried out with two independent approaches: the GUM uncertainty framework, giving an expanded uncertainty of ±926 µA/A (k = 2), and the Monte Carlo method, giving ±906 µA/A (k = 2). The good agreement between the two approaches confirms the reliability of the uncertainty budget. These results provide a useful basis for the selection of the core material and the design of a DCCT based on the tested core types.

## Figures and Tables

**Table 2:** Shunt voltage values with related standard deviations.

V_shunt_ (V)	*S*_Vshunt_ (µV)	*I*_dcp_
−23 × 10^−6^	174	−0.0003 A
0.214284	157	3.0007 A
0.357103	154	5.0006 A

**Table 3:** Uncertainty budget of the reference current measurement, expressed per ampere of the measured current.

Input Quantity	Standard Uncertainty *u*(*x*_i_)	Contribution (µA/A)
*V*_shunt_—repeatability (Type A)	154 µV	431.2
*V*_shunt_—digitizer NI 4461	60 µV	168.0
*R*_shunt_—dc calibration	0.4 µΩ	5.0
*R*_shunt_—drift	0.1 µΩ	1.0
*R*_shunt_—temperature effect	1.1 µΩ	15.0
*R*_shunt_—power effect	0.1 µΩ	0.5
*R*_shunt_—humidity effect	0.1 µΩ	1.0
*u*(*V*_shunt_)	165.3 µV	462.8
*u*(*R*_shunt_)	1.1 µΩ	15.9
Combined standard uncertainty *u*_c_(*I*_ref_)/*I*_ref_		463
Expanded uncertainty, GUM (*k* = 2)		±926
95% coverage interval, Monte Carlo (10^4^ trials)		±906

## Data Availability

The data presented in this study are available on request from the corresponding author.
